# Se-SAD serial femtosecond crystallography datasets from selenobiotinyl-streptavidin

**DOI:** 10.1038/sdata.2017.55

**Published:** 2017-04-25

**Authors:** Chun Hong Yoon, Hasan DeMirci, Raymond G. Sierra, E. Han Dao, Radman Ahmadi, Fulya Aksit, Andrew L. Aquila, Alexander Batyuk, Halilibrahim Ciftci, Serge Guillet, Matt J. Hayes, Brandon Hayes, Thomas J. Lane, Meng Liang, Ulf Lundström, Jason E. Koglin, Paul Mgbam, Yashas Rao, Theodore Rendahl, Evan Rodriguez, Lindsey Zhang, Soichi Wakatsuki, Sébastien Boutet, James M. Holton, Mark S. Hunter

**Affiliations:** 1 Linac Coherent Light Source, SLAC National Accelerator Laboratory, Menlo Park, California 94025, USA; 2 Stanford PULSE Institute, SLAC National Accelerator Laboratory, Menlo Park, California 94025, USA; 3 Biosciences Division, SLAC National Accelerator Laboratory, Menlo Park, California 94025, USA; 4 Stanford Synchrotron Radiation Lightsource, SLAC National Accelerator Laboratory, Menlo Park, California 94025, USA; 5 Department of Biochemistry and Biophysics, University of California, San Francisco, California 94158, USA

**Keywords:** Imaging, Biological physics, Nanocrystallography, Macromolecules and clusters

## Abstract

We provide a detailed description of selenobiotinyl-streptavidin (Se-B SA) co-crystal datasets recorded using the Coherent X-ray Imaging (CXI) instrument at the Linac Coherent Light Source (LCLS) for selenium single-wavelength anomalous diffraction (Se-SAD) structure determination. Se-B SA was chosen as the model system for its high affinity between biotin and streptavidin where the sulfur atom in the biotin molecule (C_10_H_16_N_2_O_3_S) is substituted with selenium. The dataset was collected at three different transmissions (100, 50, and 10%) using a serial sample chamber setup which allows for two sample chambers, a front chamber and a back chamber, to operate simultaneously. Diffraction patterns from Se-B SA were recorded to a resolution of 1.9 Å. The dataset is publicly available through the Coherent X-ray Imaging Data Bank (CXIDB) and also on LCLS compute nodes as a resource for research and algorithm development.

## Background & Summary

The LCLS at SLAC National Accelerator Laboratory was the first hard X-ray free electron laser (X-ray FEL) and was originally designed to operate up to photon energies of 8.3 keV^1^. Since the initial operation of the hard X-ray beam lines at LCLS, the maximum photon energy achieved has steadily moved higher and 11.2 keV operations were achieved. In 2015 a new method was developed to allow LCLS to operate at photon energies above the selenium K-edge of approximately 12.65 keV, paving the way for *de novo* phasing capability by using selenium single-wavelength anomalous diffraction (Se-SAD)^2^. Extending the maximum operating photon energy above the Se K-edge allows the powerful technique of Se-SAD to be used at LCLS and the traditional benefits of SFX to be exploited while collecting high-resolution data sets of novel structures.

The usual approach to Se-SAD is to substitute sulfur atoms with selenium in endogenous methionine residues for proteins that contain methionine. Given that 108,588 out of 123,622 structures (88%) in the RCSB protein data bank (www.rcsb.org) contain methionine demonstrates the potential broad applicability of this technique. In fact, SAD phasing accounted for over 70% of the novel structures deposited to the protein data bank in 2014 and Se has been used more frequently than any other element for successful experimental phasing^3^.

**Figure 1:**
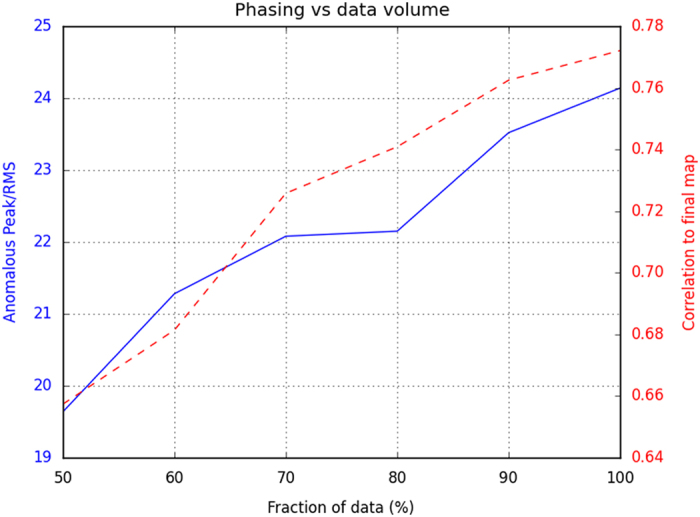
Phasing versus data volume. The correlation to final map (red) and anomalous peak RMS (blue) shown as a function of data volume used for the Se-SAD data. Using 100% of the data, only ~0.1% of the 200,000 attempts to phase the data were successful. A key to successful phasing was finding the NCS operator, which only occurred when including the entire data set. Adopted from Hunter *et al.*^2^

This paper reports the deposition of two Se-SAD SFX datasets (Data Citation 1 and Data Citation 2) acquired at LCLS as reported in Hunter *et al.*^2^ Analysis showed that although weak anomalous differences were measured to 1.9 Å, the data could be used to successfully phase the structure from a selenobiotinyl-streptavidin co-crystal. We show the correlation to final map and anomalous peak as a function of data volume used in Fig. 1. We were not able to successfully phase using a subset of the dataset. However, we hope this dataset will prove to be useful for the crystallography community to continue research and improve analysis packages with the goal of reducing the number of diffraction patterns required.

## Methods

### Overview

**Figure 2:**
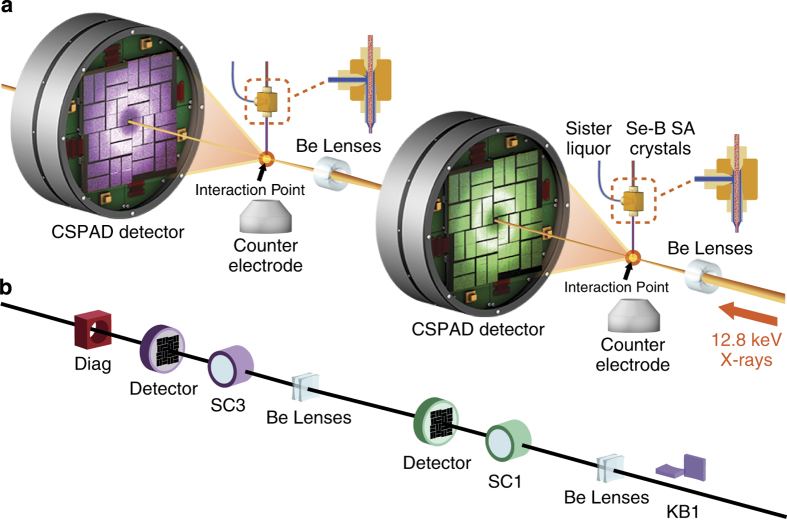
Overview of the Selenium single-wavelength anomalous diffraction experiments. (**a**) Data were collected simultaneously in two sample chambers using a serial SFX setup. (**b**) The X-rays are focused only using Be lenses with the Kirkpatrick-Baez mirrors (KB1) moved out. The X-rays enter the first sample chamber (SC1) and scatter from the Se-B SA crystal. The unscattered X-rays exit through the central hole in the CSPAD detector, which is then refocused for another scattering in the downstream sample chamber (SC3) followed by the diagnostic (Diag).

The sample used for the Se-SAD phasing study was streptavidin in complex with selenobiotin, in which the sulfur atom of the biotin molecule is substituted by selenium. The preparation of the crystals of selenobiotinyl-streptavidin (Se-B SA) co-crystals was described previously^2^. X-ray diffraction data were acquired at the Coherent X-ray Imaging (CXI) instrument of the LCLS^4^. The data were collected simultaneously from two separate sample injection setups running independently at CXI in a serial SFX configuration^5^, in which the unscattered beam from an upstream SFX experiment is refocused to a second, downstream experiment, each with an independent 2.3 Megapixel Cornell-SLAC Pixel Array Detector (CSPAD) camera^6^ as shown in Fig. 2. The CSPAD data, as well as data from many other detectors and process variables (PVs), were stored by the LCLS data acquisition system in Extensible Tagged Container (XTC) files^7^. A subset of PVs contained in the XTC files that were used during the analysis is listed in Table 1.

### Sample preparation and injection

**Figure 3:**
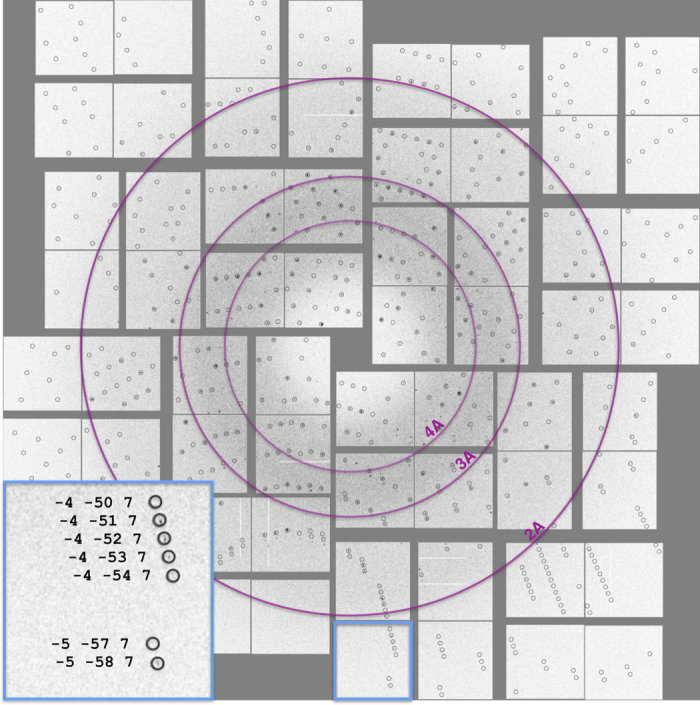
Diffraction pattern with predicted Bragg peak positions. Inset shows a zoomed in subpanel beyond the 2 Angström resolution. Adopted from Hunter *et al.*^2^

For the crystallization experiments, lyophilized, recombinant, high-purity core-streptavidin protein was purchased from Creative Biomart (Cat# Streptavidin-501) and selenobiotin was purchased from Santa Cruz Biotechnology (Cat # sc-212,920). For crystallization screening, selenobiotin was mixed in a 2:1 molar ratio with 25 mg ml^−1^ streptavidin in 22.5% (v/v) 2-methyl-2,4-pentanediol and incubated on ice overnight. The mixture was centrifuged at 14,000 g for 10 min to separate and discard solid impurities. The final mixture was screened against a library of 3,000 crystallization conditions by combining equal volumes of protein with each crystallization condition in 72 well-format Terasaki microbatch plates, covering with 100% paraffin oil, and storing at room temperature. Initial results were evaluated using a light microscope and a limited number of conditions were selected for further optimization and evaluation. Crystals from promising conditions were screened for diffraction quality at beamline 12-2 of the Stanford Synchrotron Radiation Lightsource. Diffraction patterns collected at CXI from crystals grown in 24% PEG 1,500 and 20% glycerol routinely extended to a resolution beyond 2 Å (Fig. 3).

### Sample introduction

A coMESH injector was setup for each sample chamber as described previously^8^ but was modified to fit in the standard CXI injector setup, having the tee outside of vacuum and a 1.5 m long concentric length of capillaries, reaching the interaction region. The inner sample line was 2 m of 100*×*160 *μ*m fused silica capillary directly connected to custom made LCLS sample holders. The concentric capillary was 250×360 μm fused silica capillary. Up to 5 kV voltage (less than 1 μA current) was applied to the sister liquid (The sister liquor was the same MPD sister liquor reported in Sierra *et al.*^8^). The flowrate of the sister liquor was adjusted between 1–10 μl min^−1^ to achieve stable sample introduction.

### Transmission series

Diffraction data were collected at three transmission settings, with approximate pulse energies of 0.93, 0.46, and 0.093 mJ corresponding to transmission of 100, 50, and 10%, respectively. Lower pulse energy was used to ensure accurate measurements of the low-resolution reflections. The pulse energy at the sample was controlled independently from the accelerator using Si attenuators upstream on the experiment. The PVs for the attenuators can be found in Table 1 along with the description; a value of ~0 mm indicates that the attenuator is in the beam path whereas values of approximately −20 mm indicate the attenuator is out of the beam path. In order to independently determine the transmission of the X-rays, the total thickness of the Si attenuators in the beam path should be calculated and then the transmission of the X-rays through that thickness of Si can be calculated using the center for X-ray Optics (CXRO) database^9^.

The pulse energy of the X-rays downstream of the undulators can be found on a shot by shot basis by extracting one of the six PVs from the XTC (or converted HDF5 files) associated with the readouts from the LCLS gas detectors, with the PVs listed in Table 1. The gas detectors are located in the front-end enclosure (FEE) and two of the gas detectors are located upstream of the FEE gas and solid attenuators, whereas two are downstream of the attenuators; two duplicate measurements are made downstream of the FEE attenuators with 10% of the dynamic range of the main detectors and can be used for experiments with low pulse energies. For the datasets described in this manuscript, no beam attenuation was done in the FEE and therefore the upstream and downstream gas detectors will have very similar pulse energy measurements.

### Hit finding

Raw data collected at LCLS were processed using a hit finding software called Cheetah^10^. A total of 1,567,793 diffraction patterns were identified as potential crystal hits (Table 2). The peak finding parameters for the front and the back sample chambers are summarized in Tables 3 and 4.

### Indexing

The detector distances for the upstream and downstream experiments were read from EPICS PVs CXI:DS1:MMS:06:RBV and CXI:DS2:MMS:06:RBV, respectively, but the distances (in mm) are related to the CXI beamline configuration. To convert the detector stage PVs to working distance (the physical distance between the sample interaction plane and the detector face), detector offsets need to be added to the PVs. For the upstream experiment, the working distance is determined by adding CXI:DS1:MMS:06:RBV to the detector offset of 583 mm. For the downstream experiment, the working distance is determined by adding CXI:DS2:MMS:06:RBV to the detector offset of 315 mm.

Data from both chambers and all pulse energies/transmissions were combined into the final data set, with saturated peaks being rejected from the integration process. CrystFEL peak search was used to index the crystal hits. Based on the initial indexing results, we determined that the space group was P2_1_ with a=50.7 Å, b=98.4 Å, c=53.1 Å and β=112.7°. Given the target unit cell, the indexing results were accepted if the unit cell lengths and angles were within 5% and 1.5°, respectively. The final iteration yielded 559,194 (36%) indexed patterns, with a representative pattern shown in Fig. 3. Patterns with high median background (>1,500 ADU) at low scattering angles were subsequently rejected. The remaining 481,079 (31%) patterns were then merged with *process_hkl* by only considering unsaturated peaks and reflections with more than 7 partial measurements, followed by intensity scaling (Table 5).

### Code availability

Cheetah^10^ and CrystFEL^11,12^ are free and open source software distributed under the GNU General Public Licence version 3 (GPL3), and may be downloaded from the following web locations: https://www.desy.de/~barty/cheetah and http://www.desy.de/~twhite/crystfel.

## Data Records

We have deposited two Se-SAD datasets (Data Citation 1 and Data Citation 2). The two datasets are from the two sample chambers associated with LCLS experiment names cxic0415 and cxic0515, respectively. We have deposited the raw XTC files generated by the LCLS data acquisition system, without any processing. XTC files are the native format of LCLS can be read using analysis frameworks^7^ provided by the LCLS (see https://confluence.slac.stanford.edu/display/PSDM/LCLS+Data+Analysis). An SFX processing program called Psocake can be used to analyse XTC files and the tutorial is located here: https://confluence.slac.stanford.edu/display/PSDM/Psocake+SFX+tutorial. We have also deposited CXI files which consists of only the patterns classified as ‘hits’ by Cheetah. This is a standard format in this field based on the Hierarchical Data Format, version 5 (HDF5). Detector calibration has been applied including pedestal correction and gain correction and the multi-panel CSPAD detector images are saved in an unassembled format. In addition to the two datasets, we have also shared Supplementary Data such as pedestals, bad pixel maps, pixel masks, spreadsheets describing each of the runs, and the lab coordinates of the pixels defined in the CrystFEL geometry files that can be used to assemble the detector panels into a geometrically correct two-dimensional image. The CrystFEL geometry files are described fully in the CrystFEL documentation found at http://www.desy.de/~twhite/crystfel.

## Technical Validation

**Figure 4:**
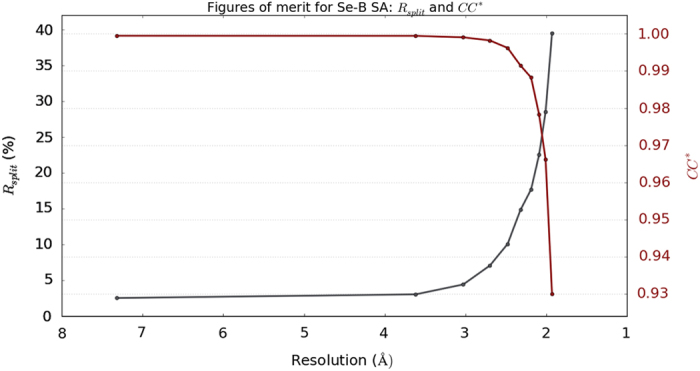
Figures of merit plot: CC* (red) and Rsplit (blue) versus resolution. CC* is an estimate of the cross correlation between the observed dataset against the unmeasured true intensities which is above 90% for our dataset up to the observed resolution shell. Rsplit is a measure of discrepancy of the measured intensities and it stays below 40% for our dataset up to the observed resolution shell. Both plots indicate the merged intensities are of high quality.

The dataset has been validated by checking for self-consistency in merged intensities. We calculated the standard figures of merit for SFX data (R_split_, CC*, CCano and I/sigma(I)) which are summarized in Table 6. Plots of R_split_ and CC* against resolution show the merged intensities are of high quality (Fig. 4). We have also shown that structure determination is possible using Se-SAD^2^. The four selenium sites were found using *phenix.hyss*^13^. The final structure produced an *R*_work_=16.6% and *R*_free_=19.9% and the electron density map (2Fo-Fc) showed the presence of strong Se peaks. The structure has been deposited in the protein data bank (ID: 5JD2).

## Additional Information

**How to cite this article:** Yoon, C. H. *et al.* Se-SAD serial femtosecond crystallography datasets from selenobiotinyl-streptavidin. *Sci. Data* 4:170055 doi: 10.1038/sdata.2017.55 (2017).

**Publisher’s note:** Springer Nature remains neutral with regard to jurisdictional claims in published maps and institutional affiliations.

## Supplementary Material



## Figures and Tables

**Table 1:** Description of the subset of recorded PVs that were used during the analysis of the Se-SAD data.

**Process Variable (PV)**	**Units**	**Description**
GDET:FEE1:241:ENRC	mJ	Pulse energy measurement at an upstream gas detector
GDET:FEE1:242:ENRC	mJ	Second pulse energy measurement at an upstream gas detector
GDET:FEE1:361:ENRC	mJ	Pulse energy measurement at a downstream gas detector
GDET:FEE1:362:ENRC	mJ	Second pulse energy measurement at a downstream gas detector
GDET:FEE1:363:ENRC	mJ	Duplicate measurement as 361 with 10% dynamic range
GDET:FEE1:364:ENRC	mJ	Duplicate measurement as 362 with 10% dynamic range
CXI:DS1:MMS:06:RBV	mm	Upstream detector stage readback value
CXI:DS2:MMS:06:RBV	mm	Downstream detector stage readback value
XRT:DIA:MMS:02:RBV	mm	20 μm thick Si attenuator motor
XRT:DIA:MMS:03:RBV	mm	40 μm thick Si attenuator motor
XRT:DIA:MMS:04:RBV	mm	80 μm thick Si attenuator motor
XRT:DIA:MMS:11:RBV	mm	160 μm thick Si attenuator motor
XRT:DIA:MMS:06:RBV	mm	320 μm thick Si attenuator motor
XRT:DIA:MMS:07:RBV	mm	640 μm thick Si attenuator motor
XRT:DIA:MMS:08:RBV	mm	1,280 μm thick Si attenuator motor
XRT:DIA:MMS:09:RBV	mm	2,560 μm thick Si attenuator motor
XRT:DIA:MMS:10:RBV	mm	5,120 μm thick Si attenuator motor

**Table 2:** Number of diffraction patterns extracted from front and back chambers.

**Transmission**	**Number of frames (Front/Back)**	**Number of hits (Front/Back)**	**Number of indexed (Front/Back)**
100%	2,520,580/1,174,470	324,803/369,691	125,755/88,289
50%	1,026,168/1,109,319	239,442/377,793	115,993/158,865
10%	1,041,249/730,055	111,444/144,620	43,188/27,104
Total	7,601,841	1,567,793	559,194

**Table 3:** Cheetah hit finding parameters in the front chambers.

**Transmission**	**Min. peaks**	**Peak criteria**	**Peak search radii (pixels)**	**Cheetah peakfinder algorithm**	**Background subtraction for peak search**
**100%**	5	Threshold 150, Peak size 3–12, SNR 6	0–1,300	8	Radial background subtraction
**50%**	10	Threshold 150, Peak size 3–12, SNR 4	0–1,300	8	Radial background subtraction
**10%**	10	Threshold 150, Peak size 3–12, SNR 4	0–1,300	8	Radial background subtraction

**Table 4:** Cheetah hit finding parameters in the back chambers.

**Transmission**	**Min. peaks**	**Peak criteria (Back)**	**Peak search radii (pixels)**	**Cheetah peakfinder algorithm**	**Background subtraction for peak search**
**100%**	10	Threshold 150, Peak size 3–15, SNR 4	0–1,300	8	Radial background subtraction
**50%**	10	Threshold 150, Peak size 3–12, SNR 4	0–1,300	8	Radial background subtraction
**10%**	10	Threshold 150, Peak size 3–15, SNR 4	0–1,300	8	Radial background subtraction

**Table 5:** CrystFEL processing parameters.

	**Peak search method**	**Peak search parameters**	**Radii of integration (pixels)**	**Minimum measurements**
**Front chamber**	CrystFEL (‘zaef’)	Threshold 500, min grad 500,000, SNR 5.5	3.5,5,5.5	7
**Back chamber**	CrystFEL (‘zaef’)	Threshold 550, min grad 1,100,000, SNR 5	3,4,5	7

**Table 6:** Selenobiotinyl streptavidin crystallography figures of merit.

	**Selenobiotinyl Streptavidin**
**Data collection**	PDB ID (5JD2)
Beamline	LCLS (CXI)
Space group	P2_1_
Cell dimensions	
*a*, *b*, *c* (Å)	50.7, 98.4, 53.1
α, β, γ (°)	90, 112.7, 90
Resolution (Å)	32.51–1.90 (1.97–1.90)
*R*_split_	0.048 (0.395)
*I*/σ(*I*)	14.0 (2.7)
Completeness (%)	1.0 (1.0)
SFX multiplicity of observations	1447.6 (1003.3)
CC*	1.000 (0.930)
CC^1/2^	0.998 (0.762)
CC^ano^	0.177 (0.003)
Wilson B Factor (Å^2^)	29.58
Refinement	
No. reflections	38,327 (3,817)
*R*_work_/*R*_free_	0.166/0.199 (0.231/0.253)
Ramachandran favored (%)	89.3
Ramachandran allowed (%)	10.5
Ramachandran outliers (%)	0.2
No. atoms	
Protein	3,630
Ligand/Ion	64
Water	265
*B*-factors (Å^2^)	
Protein	34.0
Ligand/Ion	38.9
Water	43.5
R.m.s. deviations	
Bond lengths (Å)	0.006
Bond angles (°)	1.04
